# Embedded macrophages induce intravascular coagulation in 3D blood vessel-on-chip

**DOI:** 10.1007/s10544-023-00684-w

**Published:** 2023-12-12

**Authors:** H.H.T. Middelkamp, H.J. Weener, T. Gensheimer, K. Vermeul, L.E. de Heus, H.J. Albers, A. van den Berg, A.D. van der Meer

**Affiliations:** 1 BIOS lab-on-a-chip group, University of Twente, Enschede, the Netherlands; 2 Applied Stem Cell Technologies, University of Twente, Enschede, the Netherlands

**Keywords:** Organ-on-chip, Thromboinflammation, Vessel-on-chip, THP-1, hiPSC-EC, Macrophage

## Abstract

Macrophages are innate immune cells that prevent infections and help in wound healing and vascular inflammation. While these cells are natural helper cells, they also contribute to chronic diseases, e.g., by infiltrating the endothelial layer in early atherosclerosis and by promoting vascular inflammation. There is a crosstalk between inflammatory pathways and key players in thrombosis, such as platelets and endothelial cells – a phenomenon known as ‘thromboinflammation’. The role of the embedded macrophages in thromboinflammation in the context of vascular disease is incompletely understood. Blood vessels-on-chips, which are microfluidic vascular cell culture models, have been used extensively to study aspects of vascular disease, like permeability, immune cell adhesion and thrombosis. Blood perfusion assays in blood vessel-on-chip models benefit from multiple unique aspects of the models, such as control of microvessel structure and well-defined flow patterns, as well as the ability to perform live imaging. However, due to their simplified nature, blood vessels-on-chip models have not yet been used to capture the complex cellular crosstalk that is important in thromboinflammation. Using induced pluripotent stem cell-derived endothelial cells and polarized THP-1 monocytes, we have developed and systematically set up a 3D blood vessel-on-chip with embedded (lipid-laden) macrophages, which is created using sequential cell seeding in viscous finger patterned collagen hydrogels. We have set up a human whole blood perfusion assay for these 3D blood vessels-on-chip. An increased deposition of fibrin in the blood vessel-on-chip models containing lipid-laden macrophages was observed. We anticipate the future use of this advanced vascular *in vitro* model in drug development for early atherosclerosis or aspects of other vascular diseases.

**Graphical Abstract:**

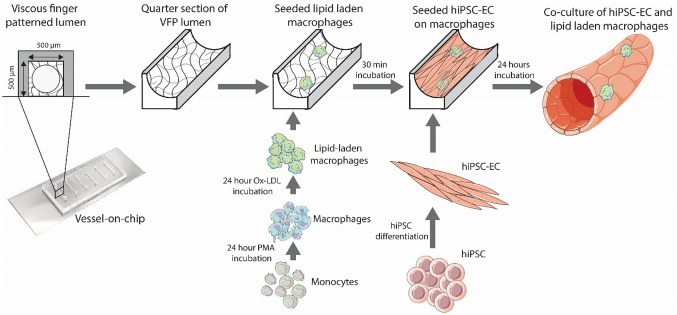

**Supplementary Information:**

The online version contains supplementary material available at 10.1007/s10544-023-00684-w.

## Introduction

Vascular diseases are the main cause of death worldwide (WHO 2019). An integral part of vascular pathophysiology is vascular inflammation. This inflammation can be induced by pathogens, vascular damage or long-term exposure of healthy blood vessels to (external) inflammatory factors such as cholesterol, toxins, and pro-inflammatory cytokines (Grootaert et al. 2018; Jia et al. 2018; Phillipson and Kubes 2011; Teague et al. 2017). Vascular inflammation leads to alterations in endothelial cell functionality, including increased expression of endothelial cell markers such as cell adhesion molecules (CAMs) and junctional adhesion molecules (JAMs) (Krieglstein and Neil Granger 2001; Weber et al. 2007). This upregulation can in turn lead to the activation and recruitment of innate immune cells from the blood stream. Innate immune cells, such as monocytes and macrophages, have a key role in wound healing, phagocytosis and in resolving vascular inflammation. These cells can be activated by an inflamed endothelial layer and are an integral part in the pathogenesis of many arterial diseases such as atherosclerosis and aneurysms (Shirai et al. 2015). After activation, monocytes can polarize towards macrophages, promoting their ability to pass through the endothelial barrier and embed underneath the endothelial layer. Macrophages have been proven to interact with endothelial cells when embedded underneath the endothelial layer (Shirai et al. 2015). These embedded macrophages can affect the endothelial cell layer by excreting either proinflammatory cytokines such as tissue necrosis factor-α (TNF- α) and interleukin-1β (IL-1β) (‘M1’ macrophages) or anti-inflammatory cytokines such as transforming growth factor-β (TGF-β) and interleukin-10 (IL-10) (‘M2’ macrophages) (Chistiakov et al. 2017). Macrophages are also known as a key players in early atherosclerosis, where polarized lipid-laden macrophages embed underneath the endothelial layer and form foam cells (Bobryshev et al. 2016; Chistiakov et al. 2017; Libby et al. 2019; Mestas and Ley 2008; Sakakura et al. 2013; Shirai et al. 2015).

Thromboinflammation is a process in which innate immune cells such as neutrophils and monocytes, interact with an inflamed or damaged endothelial layer, leading to the activation of platelets and formation of a thrombus (Rayes et al. 2020). The processes towards thrombus formation are different in arteries compared to veins, which can largely be attributed to the enormous difference in shear rates and flow (Li et al. 2019). In arteries, excreted von Willebrand factor plays a large role in platelet adhesion and accumulation, leading to recruitment of more platelets, neutrophils, and red blood cells, with fibrin threads being formed to stabilize the thrombus. Blood flow through veins can be a factor 100 lower than arterial flow, giving monocytes the opportunity to roll over the endothelial layer and therefore detect inflamed endothelial cells (Li et al. 2019; Sakariassen et al. 2015). The cell recruitment process in veins therefore differs from arteries, with monocytes instigating platelet recruitment but also being able to polarize and transmigrate through the endothelial barrier and excrete pro- or anti-inflammatory factors. Due to lower flow rates in veins and the accompanying lower shear rates experienced by endothelial cells, thrombi have a distinct histopathological presentation, typically with a high content of fibrin (Chandrashekar et al. 2018).

A relatively new method to investigate mechanisms of cardiovascular disease, including thrombosis, immune cell recruitment and endothelial inflammation are vessels-on-chips (VoCs). These microphysiological systems (MPS) are designed to recapitulate the microenvironment of blood vessels in terms of geometry, flow, extracellular matrix and cellular composition (Branchford et al. 2015; Cable et al. 2022; Ko et al. 2022; Ko and Kamm 2022; Myers and Lam 2021; Paloschi et al. 2021; Pandian et al. 2018; Song et al. 2018). VoCs typically consist of an actively perfused microfluidic channel or network of channels lined by endothelial cells. They are widely used to investigate disease processes. Multiple VoCs have already been reported that model the endothelial layer and its role in the context of thrombosis by using blood perfusion assays as an end-point measurement. (Albers et al. 2019; Barrile et al. 2018; Costa et al. 2017; van Dijk et al. 2020; Hasan et al. 2015; Jia et al. 2019; Mannino et al. 2015; Tsai et al. 2012; Westein et al. 2013; Zheng et al. 2012). Due to limitations in microfabrication, many of these VoC models have square and rectangular cross-sections, which do not mimic the human vessel structure. Advanced fabrication techniques provide the ability to make more physiologically relevant models, for example by using a viscous finger patterned hydrogel as a 3D lumen to contain the required cell types (Bischel et al. 2012; de Graaf et al. 2019; Herland et al. 2016; Miller et al. 2012). Such models can also be used to incorporate multiple cell types for studies of disease. For example, incorporation of vascular smooth muscle cells and neurovascular cells such as pericytes and astrocytes has been accomplished in multiple designs (Herland et al. 2016; Saili et al. 2018).

To study vascular inflammation in VoC models, a very important factor is the realistic incorporation of immune cells such as monocytes and macrophages. The incorporation of monocytes flowing through the blood vessel has been performed when using monocyte adhesion as an end point measurement (Halaidych et al. 2018). Even though monocyte activation, extravasation and polarization into macrophages is a long process which is hard to mimic in a VoC model, it can be an integral part of the vascular immune response. Vascular inflammation can be either induced or reduced by respectively embedded M1 and M2 macrophages. To have a physiologically relevant model and observe the cell-to-cell crosstalk in the context of vascular inflammation, and early atherosclerosis, macrophages should be incorporated directly underneath the endothelial cell layer. The complexity of a model containing human induced pluripotent derived endothelial cells (hiPSC-EC) as well as embedded (lipid-laden) macrophages, which can be kept in culture for longer periods of time and can be perfused with human whole blood has not been explored yet. By using different cell types, such as lipid-laden or non-lipid-laden macrophages, and different flow profiles, this VoC model can be used to study arterial as well as venous thromboinflammation. Such an advanced VoC model would be a key enabling technology to unravel mechanisms of thromboinflammation, potentially even in a patient-specific manner.

By performing systematic optimization of seeding properties, medium properties, and a human whole blood perfusion assay, we can now present the first blood-perfusable 3D VoC system with incorporated (lipid-laden) macrophages. We use this new 3D VoC to demonstrate that key aspects of thromboinflammation can be captured in MPS models.

## Materials and methods

### Microfluidic chip fabrication

**Fig. 1:**
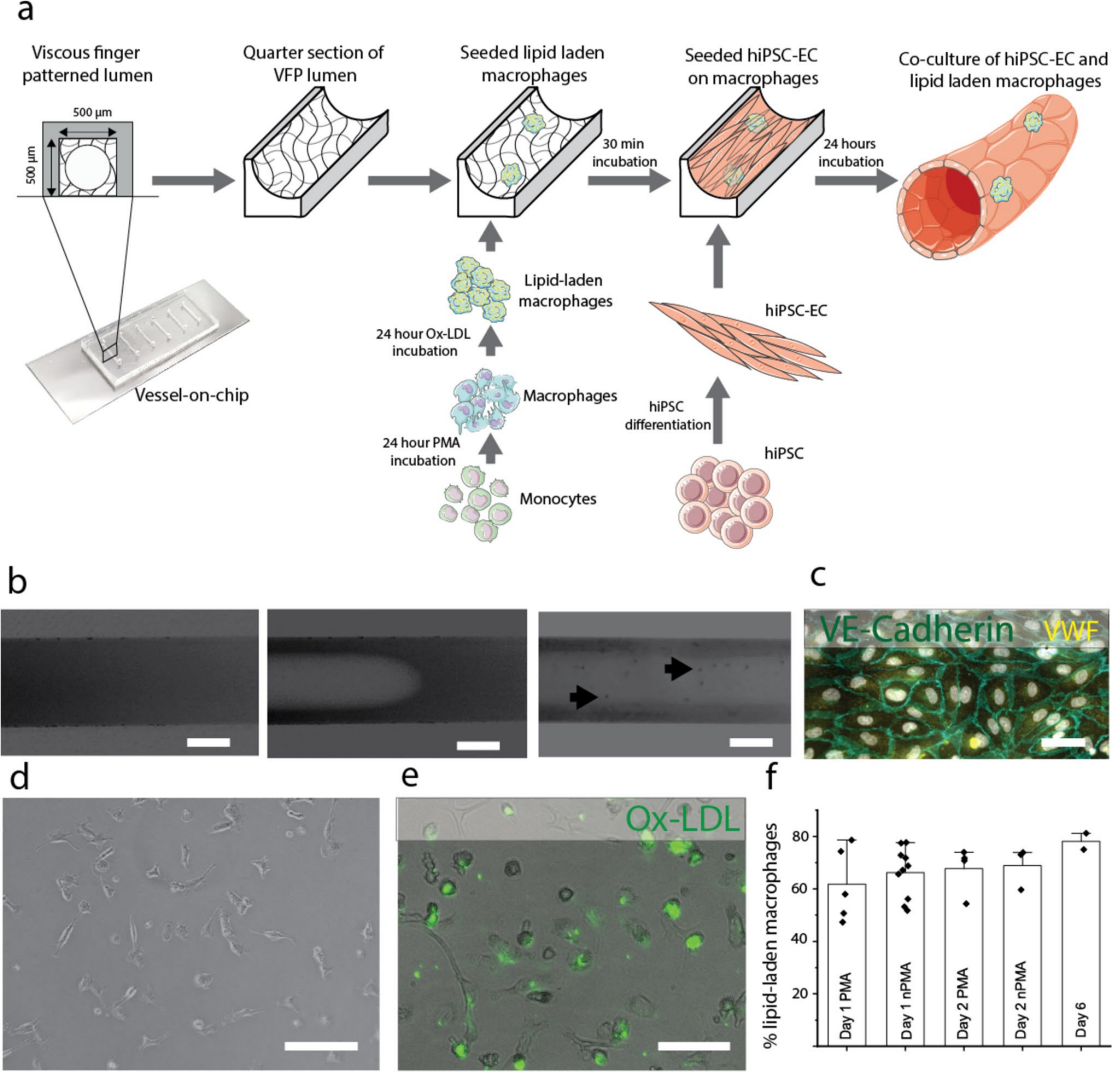
Protocol for establishing a complex 3D VoC, including characterization of its key components. **a**: Schematic overview of the protocol. 3D lumens are created in squared 500 × 500 μm collagen-filled channels. These channels are subsequently seeded with (lipid-laden) macrophages and hiPSC-EC; creating a 3D hiPSC-EC derived perfusable 3D blood vessel-on-chip with embedded macrophages. **b**: Viscous finger patterning is used to create a 3D lumen inside a channel filled with collagen type-1. Lumens can be perfused with microbeads (arrows). Scale bars represent 250 μm. **c**: Endothelial cell markers such as von Willebrand factor and vascular endothelial-cadherin (VE-cadherin) are present in hiPSC-derived endothelial cells. Scale bar represents 20 μm. **d**: Polarized THP-1 macrophages. Scale bar represents 100 μm. **e**: Polarized THP-1 macrophages can be loaded with Ox-LDL (green). Scale bar represents 100 μm. **f**: Percentage of polarized, lipid-laden macrophages as analyzed by fluorescence microscopy at multiple timepoints (‘Day 1’, ‘Day 2’, ‘Day 6’) and in the continued presence (‘PMA’) or absence (‘nPMA’) of the polarizing phorbol myristate acetate (PMA). Day 6 represents a combination of ‘PMA’ and ‘nPMA’

The microfluidic chip consists of 6 channels which are one centimeter in length and 500 × 500 μm in width and height (Fig. 1a). The chip was fabricated by conventional polydimethylsiloxane (PDMS)-based soft lithography using a poly(methyl methacrylate) (PMMA, Arkema innovative chemistry) mould. The mould was produced by micromilling (Sherline, model 5410) based on designs in SolidWorks (Dassault Systèmes, France). PDMS (10:1 base:crosslinker ratio, Sylgard 184) was added to the PMMA mould and was left to cure overnight at 65 °C after which it was removed from the mould. 1 mm inlet and outlet holes were created using biopsy punchers (Robbins Instruments). PDMS was spin coated (SPS Spin150) on a glass microscope slide and PDMS was left to cure overnight at 65 °C. The surfaces of both the microfluidic device and the microscope slide were activated by exposing them to air plasma (50 W) for 40 s (Cute, Femto Science, South Korea), after which the microfluidic chip was bonded to the microscope slide. The activated chip was then used for viscous finger patterning.

### Viscous finger patterning

The plasma-activated chips were coated with poly-dopamine (Sigma Aldrich) by adding 2 mg/ml poly-dopamine in 10 mM TrisHCl (pH 8.5) to the channels. Chips were incubated at room temperature for an hour and quickly rinsed 3 times using sterile Mili-Q water and dried. A 5 mg/ml rat tail collagen type-1 (Corning™) solution was prepared on ice, by diluting the collagen with 1/10th of the final volume of 10× PBS, supplemented with dH_2_O and NaOH to obtain a pH of 6.5–7.5. The mixture was vortexed and spun down and used within 15 minutes after mixing. 3D lumens were created in the microfluidic channels using viscous finger patterning (Bischel et al. 2012; de Graaf et al. 2019; Herland et al. 2016). A 7 mm high pipette tip was added to the outlet of the channels. 10 μl of collagen solution was pipetted in the inlet, until a meniscus formed on the outlet tip. A droplet of 2.2 μl of ice-cold PBS solution was then pipetted onto the collagen meniscus, forming a finger-like structure inside the hydrogel (Fig. 1b). Chips were incubated at 37 °C and 5% CO_2_ for one hour, after which pipette tips were removed and new pipette tips were added to the inlet and outlet. Further steps were performed using gel loader tips inside the pipette tips to not disturb the collagen lumens. 100 μl Endothelial Cell Growth medium 2 (EGM-2, PromoCell Inc.) was added to the channels and chips were incubated for 24 hours at 37 °C and 5% CO_2_ to equilibrate, before cell seeding.

### Cell culturing of hiPSC-derived endothelial cells and THP-1 monocytes

#### hiPSC-EC

Human induced pluripotent stem cell-derived endothelial cells were derived from hiPSC using an adapted previously described protocol (Orlova et al. 2014), by first performing a differentiation of the monolayer of hiPSC by inducing mesoderm using B(P)EL supplemented with CHIR (8 μM, Axon Medchem), followed by vascular specification using B(P)EL supplemented with VEGF (50 μg/ml, Miltenti) and SB431542 (10 μM, R&D Systems). Endothelial cells were isolated using CD31+ Dynabeads (Thermo fisher). hiPSC-EC were then cultured in pre-coated collagen culture flasks (Greiner bio one) at 37 °C and 5% CO_2_ using Endothelial cell serum free medium (EC-SFM (Gibco)), supplemented with 1% platelet-poor plasma-derived serum (Biomedical Technologies), 30 ng/ml VEGF (R&D systems) and 20 ng/ml bFGF (Miltenyi Biotec) (Orlova et al. 2014). To confirm the successful differentiation toward endothelial cells, the cells were stained for EC markers von Willebrand factor and VE-Cadherin (Fig. 1c).

#### Monocytes

THP-1 monocytes (ECACC) were cultured in RPMI 1640 + GlutaMAX medium (Gibco) supplemented with 1% penicillin/streptomycin (Gibco) and 10% FBS (Thermo Fisher) at 37 °C and 5% CO_2_. Cells were used up to passage 25.

#### Lipid-laden macrophages

To polarize monocytes towards macrophages, cells were seeded in a 6-well plate at 4·10^5^ cells/well and cultured overnight in serum-free RPMI 1640 medium supplemented with 50 ng/ml phorbol myristate acetate (PMA, Sigma). Fresh serum-free medium supplemented with 50 ng/ml PMA and 50 μg/ml Oxidized-LDL (DiI-OxLDL from human plasma, Invitrogen) was added after 24 hours and cells were incubated for 24 hours.

### Cell seeding in microfluidic chips

After overnight incubation of chips, cells were seeded sequentially, starting with the (lipid-laden) macrophages. Macrophages were removed from the well plate using a cell scraper and centrifuged for 5 minutes at 100×*g*. Cells were counted using a cell counter (Luna™ automated cell counter (brightfield cell counter)) and seeded in the channel at 1·10^5^ cells/ml. To make sure cells attached to the top of the channel, the microfluidic chips were incubated invertedly for 15 minutes at 37 °C and 5% CO_2_ after cells were added to the channel, after which chips were placed upright and incubated at 37 °C and 5% CO_2_ for another 15 minutes.

hiPSC-EC were seeded at passage number 3. To seed the hiPSC-EC, cells were removed from the cell culture flask, by incubating cells with 3 ml TrypLE (Gibco) for 3 minutes at 37 °C and 5% CO_2_. TrypLE was inactivated by adding 7 ml of cell culture medium to the flask. Cells were centrifuged for 5 minutes at 300×*g* and counted. Cells were seeded at 5·10^6^ cells/ml. To assure the cell attachment to both the top and the bottom of the channel, the microfluidic chips were first inverted after cells were added to the channel for at least half an hour, after which the procedure was repeated, and microfluidic chips were incubated upright at 37 °C and 5% CO_2_ for at least 30 minutes. 150 μl EGM-2 was added to the microfluidic channels. Microfluidic chips were placed on a rocking table (10° angle, 30 sec interval; BenchBlotter™ 2D platform rocker) inside the incubator for 24 hours.

### Medium optimization in microfluidic chips

Medium optimization was performed for the co-culture of hiPSC-EC and macrophages. Cells were incubated overnight with either EGM-2 or a combination of EGM-2 and RPMI 1640. After 24 hours 4× magnification brightfield images were obtained. Two separated operators individually counted the number of sprouts per channel.

### Endothelial layer assessment

After overnight incubation, the endothelial layer integrity was assessed using three conditions: healthy endothelium (control), endothelium with embedded macrophages (sample) and TNF-α treated endothelium (inflamed). Inflamed samples were treated with 5 ng/ml TNF-α (Peprotech) for 4 hours. Cells were fixated and cells were permeabilized with 0.1% Triton X-100 solution for 10 min at room temperature. Cells were incubated with Nucblue (nuclei, Thermo fisher) and actinRed (Thermo fisher). Immunocytochemistry imaging data was collected using a Zeiss LSM 880 confocal microscope at 10× magnification.

### Blood perfusion assay

Blood was provided by the Experimental Centre for Technical Medicine (ECTM, Techmed Centre, University of Twente) and was used within 4 hours of blood draw. Whole blood was collected in vacuvette tubes, containing 3.2% citrate. The first tube was always discarded. Platelets and fibrin were stained with respectively CD41-FITC (40 μl/ml; Beckmann-Coulter) and conjugated fibrinogen (10 μg/ml; Fibrinogen from human plasma, Invitrogen) for 10 minutes. Immediately before perfusion, blood was recalcified using a recalcification buffer containing HEPES; 63.2 mM CaCl_2_ (1 M stock, Thermo fisher) and 31.6 mM MgCl_2_ (Thermo fisher).

The pipette tip at the outlet was removed and replaced by a bent 14-gauge blunt needle to which tubing (Tygon) was connected, which in turn was connected to a syringe on a syringe pump (Harvard PHD Ultra). Blood was pulled through the channel at 6 μl/min for 20 minutes. After perfusion, channels were flushed manually with EGM-2 and fixated with 4% formaldehyde for 15 minutes and subsequently washed 3 times with PBS and imaged for data analysis.

### Staining and imaging

After fixation, cells were permeabilized with 0.1% Triton X-100 solution for 10 min at room temperature. Cells were incubated with Nucblue (nuclei, Thermo fisher) and actinRed (Thermo fisher) or actinGreen (Thermo fisher).

Immunocytochemistry imaging data was collected using a Zeiss LSM 880 confocal microscope at 10× magnification. Images for data analysis after blood perfusion were obtained using an EVOS M5000 imaging system (Thermo Fisher) at 4× magnification.

### Data analysis

To calculate the fibrin and Ox-LDL area coverage percentage, the thresholding function in FIJI image analysis software (Schindelin et al. 2012) was used. To avoid human error, two operators individually performed a blind assessment of the coverages and values were then compared and averaged. A paired sample t-test was performed for both the area coverage and clot size calculations.

The scoring of endothelial cell layer was performed by two operators, who individually observed endothelial cell layers and scored them from 1 (very bad) to 5 (very good). While scoring the endothelial layer, operators assessed cells morphology, monolayer formation and holes.

### Generation of figures

3D images were generated using Imaris software (Oxford Instruments). Parts of the figure were drawn by using pictures from Servier Medical Art. Servier Medical Art by Servier is licensed under a Creative Commons Attribution 3.0 Unported License (https://creativecommons.org/licenses/by/3.0/).

## Results and discussion

### 3D blood vessels-on-chip

To achieve a VoC model with embedded macrophages, we followed the scheme in Fig. 1a. 3D lumens were created in a collagen I hydrogel in a PDMS chip. These chips were acclimated in an incubator overnight before cell seeding. (Lipid-laden) macrophages were seeded, followed by hiPSC-EC. After 24 hours of incubation on a rocking table, a functional 3D VoC with embedded macrophages was achieved. To attain this model, multiple optimization steps were performed.

Firstly, circular lumen-shaped channels in a square PDMS chip by using viscous finger patterning in a collagen 1 hydrogel had to be created (Fig. 1b). Viscous finger patterning of lumens in collagen is a difficult process due to manual handling, but by standardizing the protocol, high success rates can be achieved (de Graaf et al. 2019). The most important parameters to optimize were the PBS droplet size and NaOH concentration to effectively drive flow upon viscous finger patterning and to promote proper gelation after patterning, respectively.

**Fig. 2:**
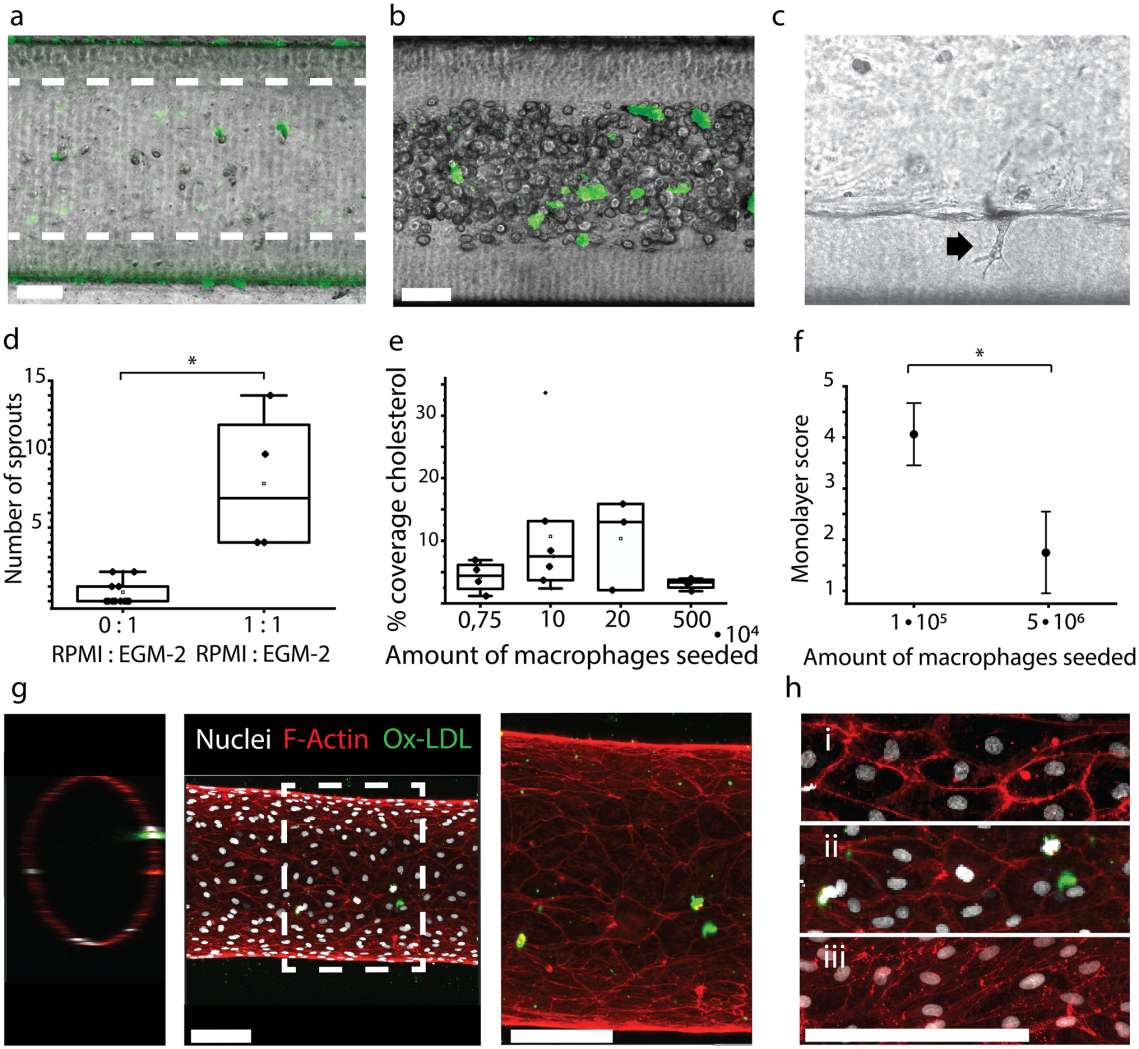
Embedded lipid-laden macrophages in 3D blood vessel-on-chip. **a**: Collagen lumen (dashed lines) loaded with lipid-laden macrophages (green Dil-Ox-LDL) seeded at 1·10^5^ cells/ml. **b**: After lipid-laden macrophages were incubated for 30 minutes, hiPSC-EC were seeded to the channel at 5·10^6^ cells/ml. Lipid laden-macrophages are visibly embedded underneath the hiPSC-EC suspension (green Dil-Ox-LDL). **c**: Example of endothelial cell sprouting. **d**: Number of sprouts observed in three representative figures of a vessel lumen, in conditions with only endothelial medium (‘0:1 RPMI:EGM-2’) or a mixture of endothelial and macrophage medium (‘1:1 RPMI:EGM-2’). **e**: Percentage of the vessel area covered with green, fluorescent signal when seeding macrophages at multiple concentrations. **f**: Effect of seeding densities of macrophages on monolayer quality (‘Monolayer score’) of hiPSC-EC after 24 hours of incubation, with a bad monolayer scored as a 1 and a very good monolayer as 5. **g**: 3D confocal images show a monolayer of hiPSC-EC in the channel after 5 days of co-culture. Ox-LDL is still visibly embedded in the endothelial layer after 5 days of incubation (Nuclei: white; Actin: Red; Ox-LDL: Green). **h**: Endothelial layer assessment, with i: healthy endothelial layer (control); ii: macrophage embedded endothelial layer (sample) and iii: TNF-α treated endothelial layer (inflamed). In i and ii a cortical actin distribution can be observed, whereas iii shows an actin redistribution towards stress fibers (Nuclei: white; Actin: Red; Ox-LDL: Green). All scale bars represent 100 μm

We systematically incorporated different cell types in our 3D blood vessel-on-chip. THP-1 monocytes were polarized, and loaded with Ox-LDL (Fig. 1d, e). The lipid-laden macrophages remained stable for up to 6 days (Fig. 1f). No difference in the number of lipid-laden macrophages was observed in well-plate experiment, when loading the cells with or without the presence of PMA. After seeding the cells at 1·10^5^ cells/ml we observed dispersed cells throughout the channel (Fig. 2a, b). Macrophages polarized from THP-1 monocytes using PMA are widely used in research, but due to differences in protocols it can be challenging to arrive at a single definitive protocol. For example, PMA incubation times can vary between 3 and 72 hours, giving the cells a completely different stimulation time (Lund et al. 2016). We have stimulated the THP-1 cells for 24 hours and, without a resting period afterwards, loaded them immediately by incubating with Ox-LDL and PMA for 24 hours, before seeding them in the lumen.

Medium optimization for co-culture of hiPSC-EC and macrophages was performed combining different ratios of EGM-2 and RPMI 1640. Figure 2d shows the difference in endothelial layers when incubated in different types of medium. It was observed that when culturing cells in anything other than EGM-2, the endothelial cells were more actively migrating and started sprouting into the hydrogel (Fig. 2c, d). A significantly higher amount of sprouts was observed for cells cultured in 1:1 (RPMI 1640: EGM-2) compared to merely EGM-2 (*p* < 0.05, Fig. 2d). Therefore, it was determined that for long-term culture of the combination of macrophages and hiPSC-EC in our VoCs, individual EGM-2 was most effective. EGM-2 is a widely used endothelial cell growth medium, optimized for primary endothelial cells and has been widely used in other projects as it contains antibiotics recommended for MPS culture (Bezenah et al. 2018; Gu et al. 2017; Olmer et al. 2018; Zhou et al. 2021).

Different macrophage seeding concentrations were tested, ranging from 7.4·10^3^ to 5·10^6^ cells/ml. Interestingly, we observed no difference between these conditions in terms of LDL coverage inside the VoC after 24 hours of co-culture with hiPSC-EC (Fig. 2e). However, VoCs seeded with high concentrations of macrophages were not able to survive for more than 24 hours. This suggests a negative effect of an abundance of macrophages on the endothelial layer. Scoring the endothelial layers of VoCs containing 1·10^5^ macrophages/ml versus 5·10^6^ macrophages/ml showed a significantly better endothelial cell layer at lower macrophage seeding densities after 24 hours (*p* < 0.05, Fig. 2f), the full extent of the endothelial layer scoring system, including scoring examples can be found in Supplementary Fig. 1. Seeding the macrophages at a lower density (7.4·10^3^–2·10^5^) and subsequent seeding of hiPSC-EC at 5·10^6^ cells/ml resulted in a full monolayer, covering the whole microfluidic channel (Fig. 2g) and enabled the VoCs to be cultured for at least 5 days. Actin staining of the channels show a quiescent monolayer of cells. We show that after 5 days, channels previously seeded with lipid-laden macrophages still contain these lipid-laden macrophages in the channel (Fig. 2g).

To assess the integrity of the endothelial layer, an actin staining was performed (Fig. 2h). A healthy endothelium, presents with a cortical actin distribution, which also is known as a barrier integrity factor, whereas in an inflamed endothelial layer, actin is known to redistribute into stress fibers (Dudek and Garcia 2001; Ehringer et al. 1999; Prasain and Stevens 2009). Figure 2h shows 3 conditions that were considered in the actin assessment: healthy endothelium (Fig. 2h(i)), endothelium with embedded macrophages (Fig. 2h(ii)) and TNF-α treated (inflamed) endothelium (Fig. 2h(iii)). It can be observed that both the healthy and the macrophage embedded endothelium show cortically distributed actin on the overall endothelial layer. The inflamed, TNF-α treated endothelium, shows a redistributed actin pattern. Therefore the observed actin distribution in the macrophage embedded VoCs indicated a quiescent, integer, endothelium. To further quantify the VoCs other functional assays may be required such a barrier permeability assay or mapping endothelial inflammation markers such as CAMs. Endothelial inflammation can also be assessed functionally, by for instance a blood perfusion assay, which is discussed in the next section.

The VoC was kept on a rocking table set to a 10° angle with a 3 second interval, giving it continuous bidirectional flow, which was calculated to yield a maximum shear rate of 140 s^−1^. This bidirectional flow enabled the cells to experience some shear stress, and we achieved culture times of up to 5 days. However, bidirectional flow is not physiologically accurate in blood vessels and preferably cells experience unidirectional flow at relevant shear stresses (Wang and Shuler 2018). Recent developments show the possibility of maintaining unidirectional flow in similar blood vessel-on-chip models at relevant shear stress, which is recommended for future research (de Graaf et al. 2022a; de Graaf et al. 2022b; Wang and Shuler 2018).

### Blood perfusion assay

**Fig. 3:**
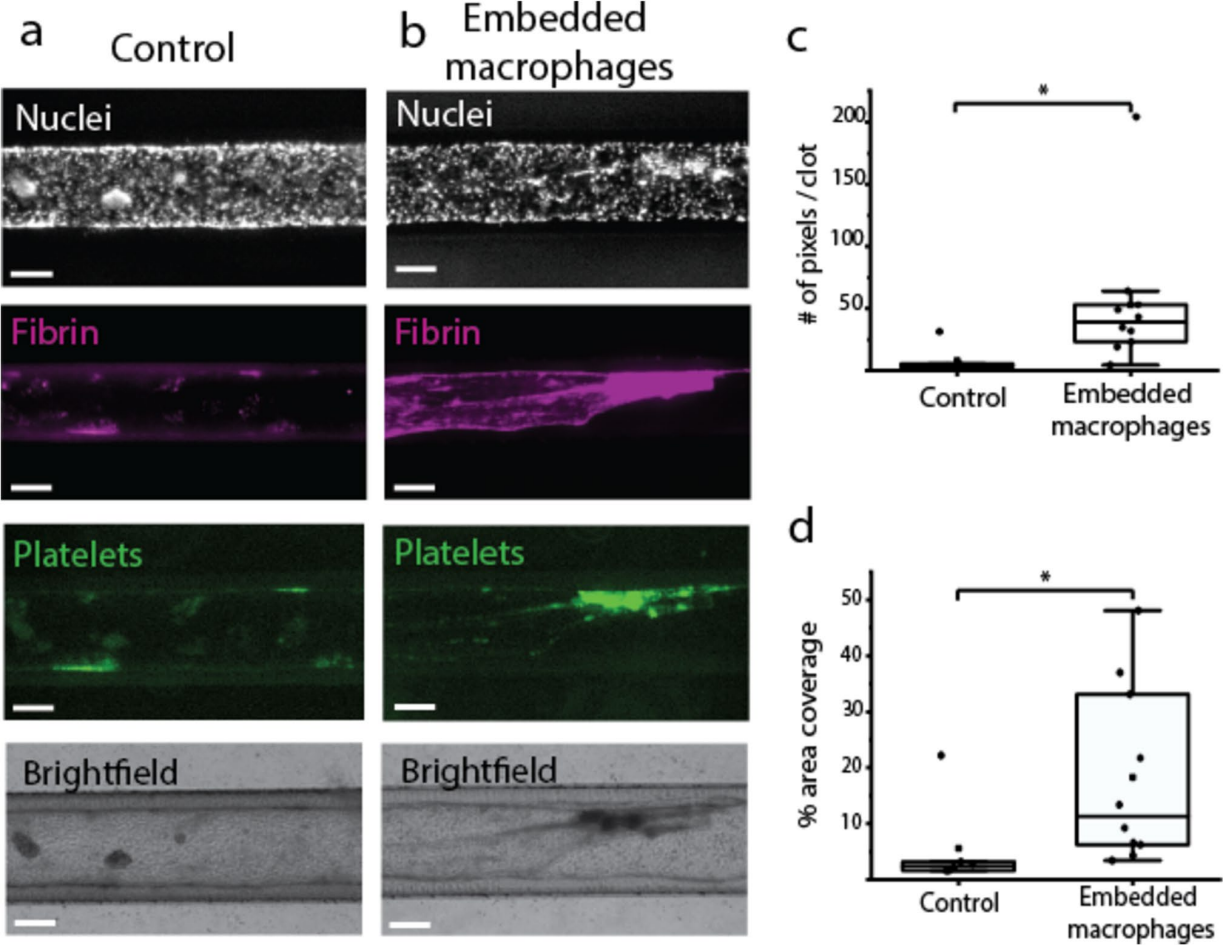
Blood perfusion assay results in a higher deposition of fibrin in the channels containing embedded macrophages; **a**: Blood vessel channel with a monoculture of hiPSC-EC (‘Control’). **b**: Blood vessel channel with co-cultured macrophages with a high % area coverage of fibrin; **c**,**d**: fibrin clot size and % area of fibrin coverage in monoculture (‘Control’) conditions and co-culture (‘Embedded macrophages) conditions. All scale bars represent 150 μm

We successfully have set up a blood perfusion assay for the 3D blood vessels-on-chip. Blood was perfused at 6 μl/min, representing a shear rate of approximately 35 s^−1^, which corresponds to the shear rate found in the human veins (Sakariassen et al. 2015). We observed a different deposition of fibrin in the blood vessels containing embedded macrophages, compared to the control samples without embedded macrophages (Fig. 3a, b). Analysis of the area covered by deposited fibrin showed a significant difference between the two groups (*p* < 0.05, Fig. 3d). Moreover, average analysis of clot size showed a significantly bigger fibrin clot size in the blood vessels containing embedded macrophages (*p* < 0.05, Fig. 3c), indicating the formation of bigger fibrin networks in this vascular inflammation model. The fibrin content in the clots is higher than what is typically seen in blood perfusion assays in vessels-on-chips with activated endothelial monocultures at high shear rates (750–1000 s^−1^) (Jain et al. 2016). This difference may reflect a more vein-like response in our model, which is expected with the flow rates used (Chandrashekar et al. 2018).

Interestingly, these results suggest the immediate negative effect of embedded macrophages on the endothelial cell layer, even without any additional external stimulation. THP-1 polarization with PMA leads to M0 macrophages, leaving them free to differentiate into either M1 or M2. The phenotype of the embedded macrophages was not determined in the current study, but future monitoring and control over this aspect could provide further insight in the model and its predictive value in disease modeling. Embedding of M1 or M2 macrophages or lipid-laden macrophages can possibly lead to a different endothelial response (Binnemars-Postma et al. 2016; Kim et al. 2018). Future studies could then focus on investigating the different effects of different types of (lipid-laden) macrophages on the endothelial layer and thromboinflammatory processes.

During the blood perfusion assay, platelet aggregation was observed. However, after the chips were fixated, washed and prepared for analysis by fluorescence microscopy, no significant difference in platelet aggregation was observed between control conditions and conditions of co-culture. Supplementary Fig. 2 shows the difference of a channel before and after preparing it for microscopic analysis. The pressure of washing clearly reduces the big platelet aggregates. To study the effect of platelet aggregation, live imaging during the blood perfusion assay would be recommended.

To obtain arterial responses and shear rates, flow rate should be increased to approximately 160 μl/min. Instantaneous high flow rate on the channels resulted in loss of cells and therefore monolayer. Optimization of the system is required if arterial rates are desired. Using unidirectional continuous flow during cell culture overnight with increasing flow rates, might assist the cells to get adjusted to the constant shear.

## Conclusion

The aim of this project was to set up a 3D vascular model containing embedded macrophages in an hiPSC-EC layer. For the first time, we have systematically set up and presented the protocol for a hiPSC-EC-loaded blood vessel-on-chip model with embedded macrophages arranged underneath the hiPSC-EC layer to study the effect of macrophages on the endothelial layer. We have successfully set up a blood perfusion assay for this vessel-on-chip model, which contains embedded (lipid-laden) macrophages and have studied the effect of these macrophages on the endothelial cell layer. Our results demonstrate a significantly higher area coverage of fibrin in the channels containing embedded macrophages, suggesting a proinflammatory effect of these macrophages and therefore endothelial response. In future studies, this blood vessel-on-chip will be used as a model to study mechanisms of vascular disease, thereby contributing to (personalized) drug development.

## Supplementary files

ESM 1
