# Supplementary material for: Embedded macrophages induce intravascular coagulation in 3D blood vessel-on-chip
Source: Biomed Microdevices. 2023 Dec 12;26(1):2. doi: 10.1007/s10544-023-00684-w (PMC10716057; doi:10.1007/s10544-023-00684-w)
Supplement: Supplementary file 1 — ESM 1 [file 10544_2023_684_MOESM1_ESM.docx]

Supplementary figures:


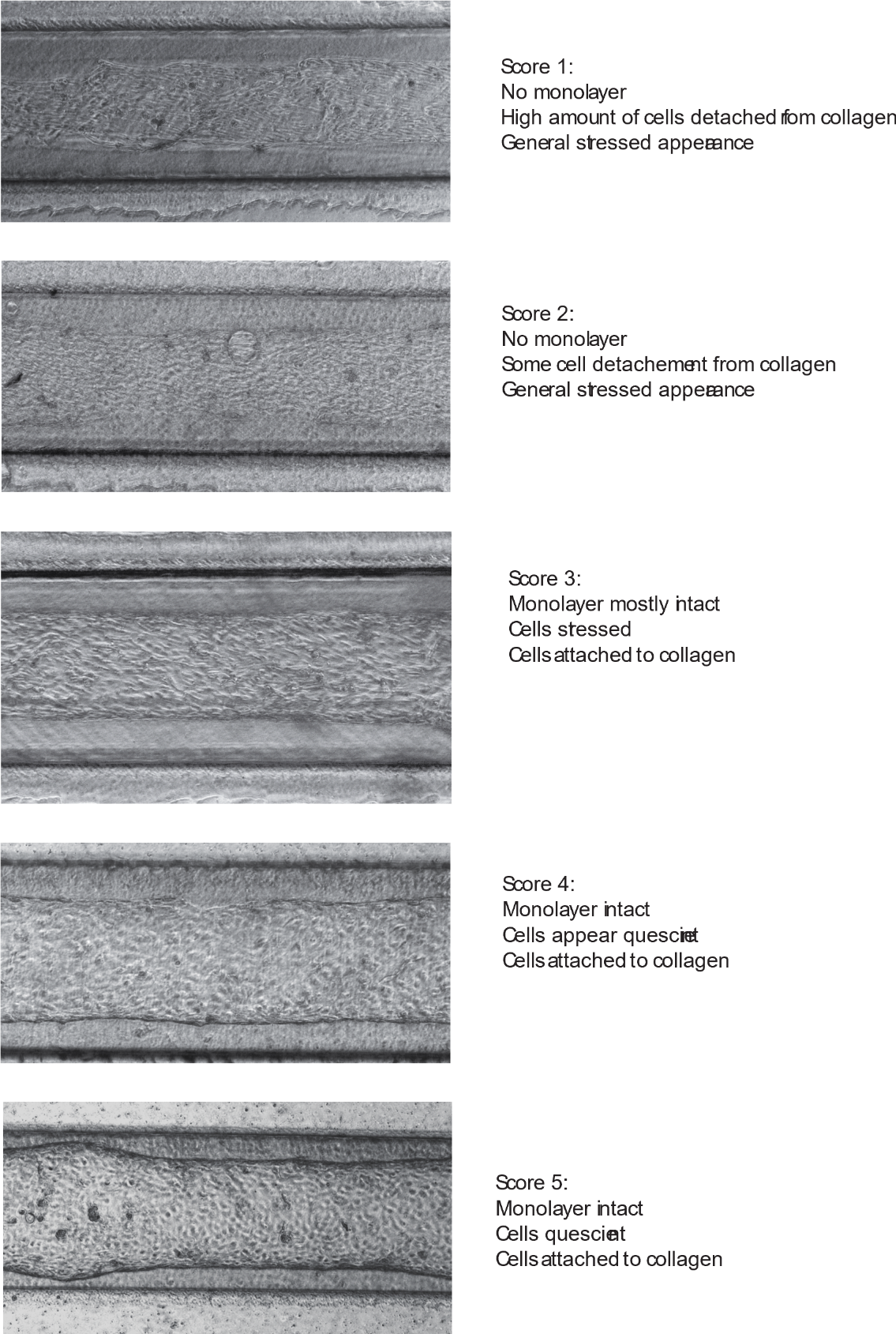


Supplementary figure 1: Cell layer scoring, cell layers were scored according to the above scoring system. A scoring of 1 meaning, there were a lot of holes in the monolayer and cells did not attach well to collagen gel and had an overall stressed appearance; a scoring of 5 shows an intact monolayer, no cells detached from the collagen gel and overall quiescent appearance of the cells.


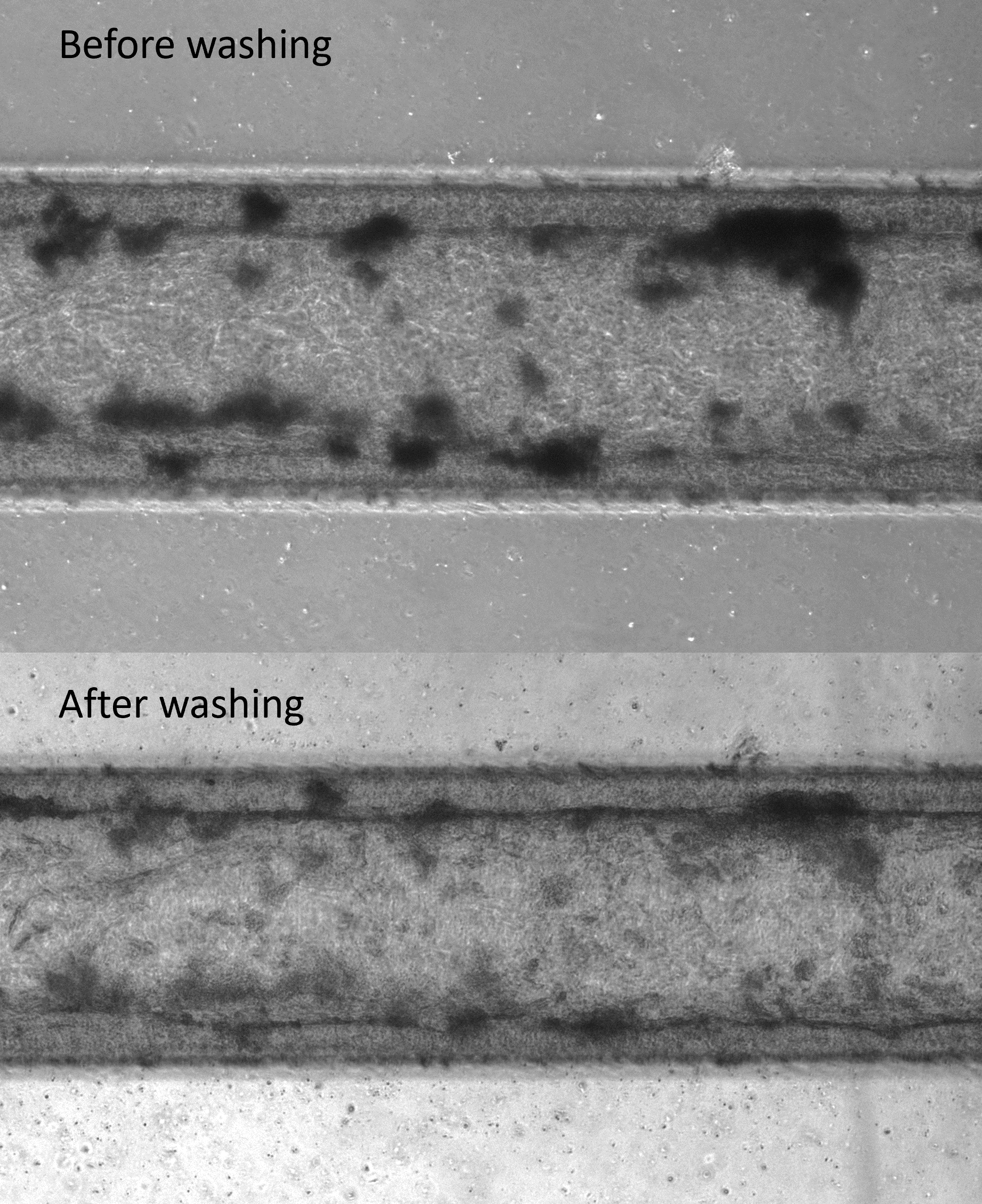


Supplementary figure 2: Washing the channel for fixation and staining, puts high amounts of pressure on the channels, therefore flushing big clots out of the channel.
